# Defining the New Poor

**Published:** 1920-04-03

**Authors:** 


### Defining the New Poor.

Manners and mankind have changed so drastically since the Great War that standards have to be revised. Before the war local authorities had a good understanding of poverty, and could easily allocate doles and concessions. This is not such an easy matter now, when those who once were poor are passing rich. Consequently, authorities are defining what persons are entitled to be classified as poor and needy. This is necessary now that local authorities are requested to see that the "necessitous" are able to get an adequate supply of milk. Hence the new poor are being scheduled, and different ideas and standards of poverty are being accepted in different localities. Faced with the problem of defining those to whom free or cheap milk should be provided, the Chelmsford medical officer of health, anxious to decide upon a fair standard of necessitousness, got in touch with the medical officers of health of Willesden, Tottenham, Edmonton, and Barking, to ascertain what was being done in those areas.

### Striking an Average.

Now he has struck an average and arrived at the subjoined classification of poverty :—

Income of family, to include (1) wages of parents, (2) earnings of children over fourteen years, (3) earnings of children under fourteen years, and (4) lodgers (reckon as income 50 per cent. of surplus over 12s. per head paid by each lodger).

Outgoing expenses. Allow weekly cost of living to all outgoing expenses inclusive per head as follows : Father, £1 ; mother, 18s. ; first child, 10s. ; other children, 8s.

Thus, for family of father, mother, and two children, allow £2 16s. as total weekly cost of living. If the income is below this standard the case is considered necessitous. Illness and other exceptional expenses are also taken into consideration.

### At Chelmsford.

After some experience of this scale, however, the Chelmsford medical officer of health is inclined to think that it might be reduced a little, and proposed to substitute 52s. for 56s. as the weekly cost of living for a family of four persons. This, he thinks, would form a satisfactory standard, adding to this sum of 52s. an additional sum of 8s. for the maintenance of each additional child beyond the number of two.

Thus the medical officer has so far attempted to deal only with necessitous cases. But the latest circular of the Ministry of Health empowers local authorities to supply milk free, or at a reduced price, not merely in necessitous cases, but also "where such a supply is necessary because of the retail price of milk in any area."

As the medical officer observes, this opens a very wide door. Even if expenditure remain the same as for the current month, it would involve a rate of $1\frac{1}{8}d.$ in the £, and if more necessitous cases are also relieved the ratepayers will have to bear a very heavy burden.

